# Risk factors of incomplete healing following medial meniscus posterior root tear repair with gracilis tendon

**DOI:** 10.1038/s41598-023-50358-z

**Published:** 2023-12-27

**Authors:** Xingen Liao, Hongbo Li, Si Nie, Min Lan

**Affiliations:** 1 Department of Orthopedics, Jiangxi Provincial People’s Hospital (The First Affiliated Hospital of Nanchang Medical College), No.92 Aiguo Road, Donghu District, Nanchang, 330006 Jiangxi Province People’s Republic of China; 2 Department of Radiology, Jiangxi Provincial People’s Hospital (The First Affiliated Hospital of Nanchang Medical College), Nanchang, 330006 People’s Republic of China

**Keywords:** Diseases, Medical research, Risk factors

## Abstract

To evaluate the clinical efficacy and meniscus healing rates of the arthroscopically assisted tendon graft fixation of the medial meniscus posterior root tears (MMPRTs), and to identify some independent risk factors correlated with meniscal root healing status. We conducted a retrospective study with 129 patients who received arthroscopically assisted tendon graft fixation of the MMPRTs between January 2018 and September 2021. Functional recovery of the knee was evaluated and meniscal root healing status was assessed. The associations between different clinical factors and meniscal root healing status were analyzed. 98 (76.0%) patients had complete meniscal root healing with a minimum 2-year follow-up, and the Lysholm score, international knee documentation committee score, and visual analogue scale score were significantly improved at final follow-up (P < 0.001; respectively). Binary logistic regression models analysis and the receiver operating characteristic curve was performed to detect independent risk factors for incomplete healing, and these results indicated that age (OR = 1.095, P = 0.039), body mass index (BMI) (OR = 1.259, P = 0.018), preoperative meniscus extrusion (OR = 5.181, P < 0.001) and varus degree (OR = 7.764, P < 0.001) were the independent risk factors correlated with incomplete healing in patients with repaired MMPRTs. In conclusion, the arthroscopically assisted tendon graft fixation of the MMPRTs can provide good clinical and radiological outcome. Additionally, we identified age > 37.5 years, BMI > 24.5 kg/m^2^, preoperative meniscus extrusion > 2.7 mm and varus degree > 3.3° as independent risk factors correlated with incomplete meniscus root healing status.

## Introduction

The meniscus is a critical component of the knee and plays an essential role in maintaining various aspects of knee functions. During the past decade, growing interest has focused on medial posterior meniscal root tears (MMPRTs), which are defined as bony or soft tissue root avulsion tears or radial tears adjacent to the attachment of the medial meniscus root^1^. Investigators have reported that the traumatic avulsed root tears associated with ligament injuries are more common in younger patients, while the degenerative root tears often occur in elder patients usually following minor trauma^2^.

Study has shown that the complete MMPRTs dramatically inhibit meniscal function similarly to total meniscectomy, thereby being strongly correlated to knee osteoarthritis Compared with partial meniscectomy, there is more evidence indicating that anatomic fixation of the MMPRTs restores both the anatomical and hoop tension of the medial meniscus, and should be recommended for young and active patients^5,6^. Transtibial pull-out repair by securing the meniscus root to its original anatomy has been reported as a promising approach to clinical improvement, however, results of MRI or second-look arthroscopy studies evaluating the repaired meniscus healing status have shown that less than 2/3 of patients have healed completely^7^.

The initial fixation strength of the suture must be strong enough to maintain the entire biological healing, therefore, Holmes et al.^8^ presented an arthroscopic reconstructive technique using gracilis autograft with suture reinforcement for MMPRTs which is expected to yield improved healing rates compared with direct repair techniques. While the risk factors for MMPRTs and prognostic factors for anatomic fixation of MMPRTs have been reported^9,10^, the risk factors correlated with the meniscal root healing status of reconstruction MMPRTs have not been elucidated in the literature.

The purpose of the present study was to evaluate the clinical efficacy and meniscus healing rates of the arthroscopically assisted tendon graft fixation of MMPRTs, and to identify some independent risk factors correlated with the healing status of meniscal root after anatomic fixation. The hypothesis was that there would be relevant factors affecting the healing status of meniscal root after anatomic fixation.

## Materials and methods

### Patient selection

After obtaining approval from the medical research ethics committee of our institution, all procedures performed in this study involving human participants were in accordance with the bioethical standards of the institutional and national research committees and with the 1964 Declaration of Helsinki and its later amendments. Diagnosis of patients with isolated MMPRTs mainly relied on knee 3.0 T magnetic resonance imaging (MRI) findings such as cleft, giraffe neck, and ghost signs^11^, with subsequent arthroscopic confirmation.

The inclusion criteria were: (1) MMPRTs on arthroscopic findings and treated with arthroscopic tendon grafts reconstruction technique, (2) knee osteoarthritis grades 0–2 Kellgren-Lawrence (K-L) And the exclusion criteria were: (1) combined cartilage resurfacing or ligament reconstruction, (2) combined corrective osteotomy , (3) prior knee surgery, (4) osteoarthritis K-L grades 3–4, (5) irreparable complex MMPRTs, (6) concomitant multiple-ligament injuries or other associated knee joint lesions, (7) follow up time less than 2 years.

### Data collection

The following parameters were recorded: demographic features, comorbidities, preoperative K-L stages knee osteoarthritis were evaluated on standing anteroposterior radiograph, hospitalization time, injury side, MMPRTs type^12^, preoperative varus degree on weight-bearing X-rays (preoperative long-leg standing radiographs were obtained from all the patients to assess hip–knee–ankle angle), preoperative medial meniscus extrusion (meniscus displacement from the superomedial aspect of the tibial plateau to the periphery of the meniscal body, at the level of the medial collateral ligament)^13^, preoperative and postoperative visual analogue scale (VAS), Lysholm score and international knee documentation committee (IKDC) score of the affected knee, complications, and radiological outcomes of the repaired MMPRTs at the final follow-up.

### Surgical technique

**Figure 1:**
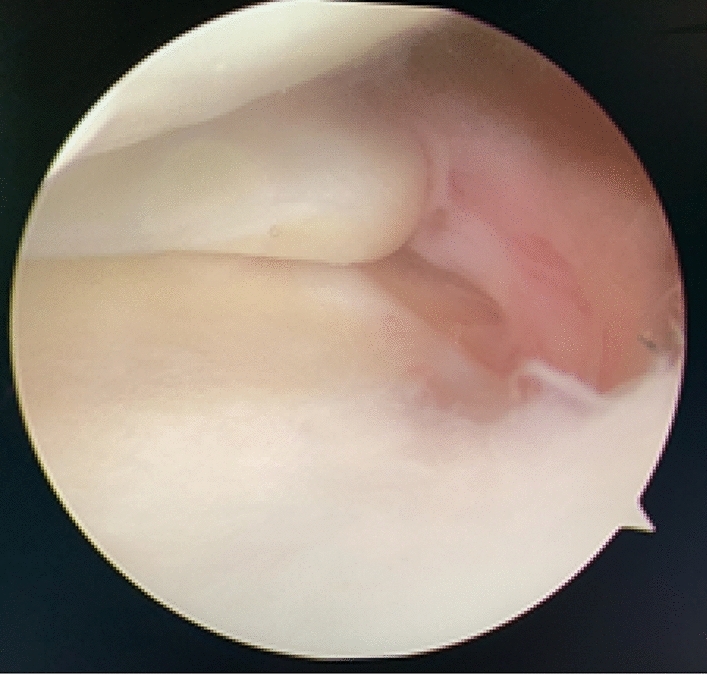
Diagnostic arthroscopy to understand the presence and extension of MMPRTs and other eventual intra-articular lesions.

**Figure 2:**
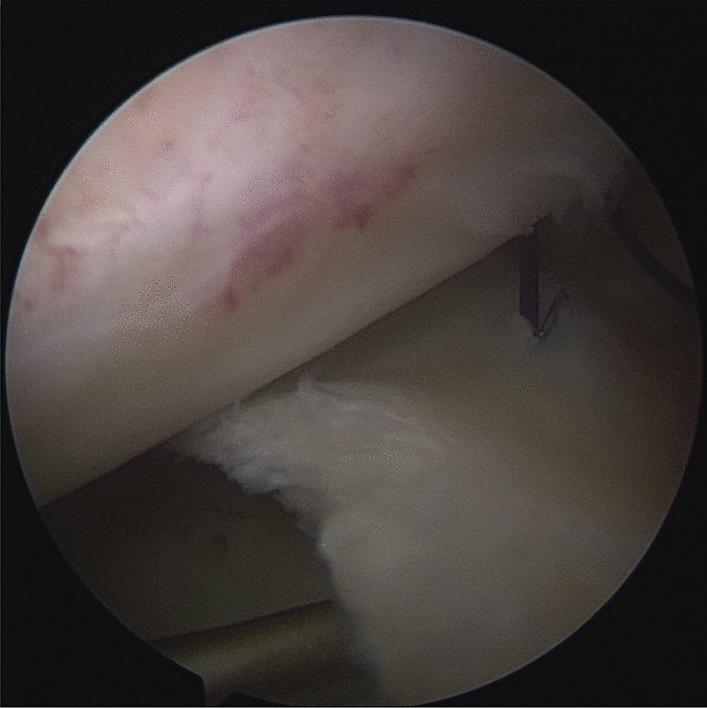
A self-passing suture device (Sharpshooter, Ivy Medical) was placed in the accessory portal to pass a No. 0 fiber wire suture (Smith & Nephew) through the posterior portion of the torn meniscus root.

**Figure 3:**
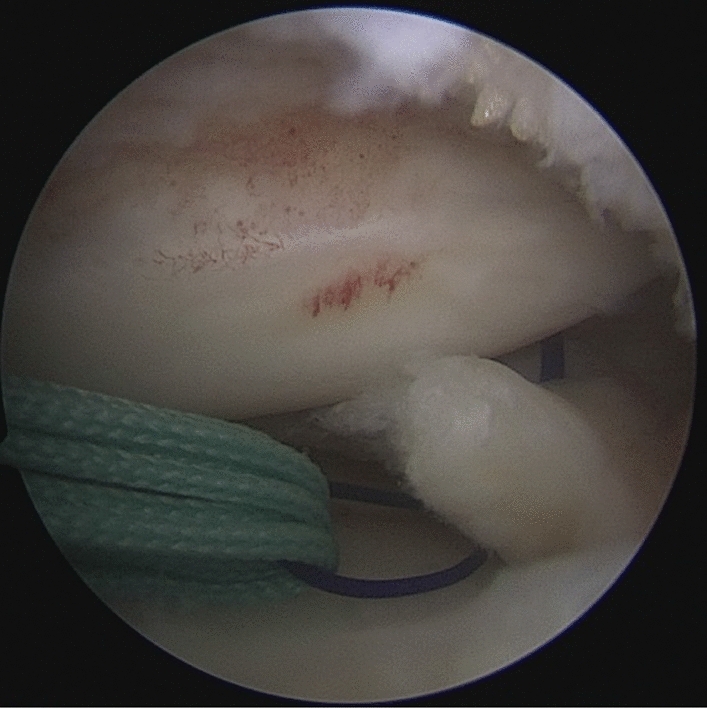
The soft tissue tunnel is dilated with multiple passes of No. 0 fiber wire.

**Figure 4:**
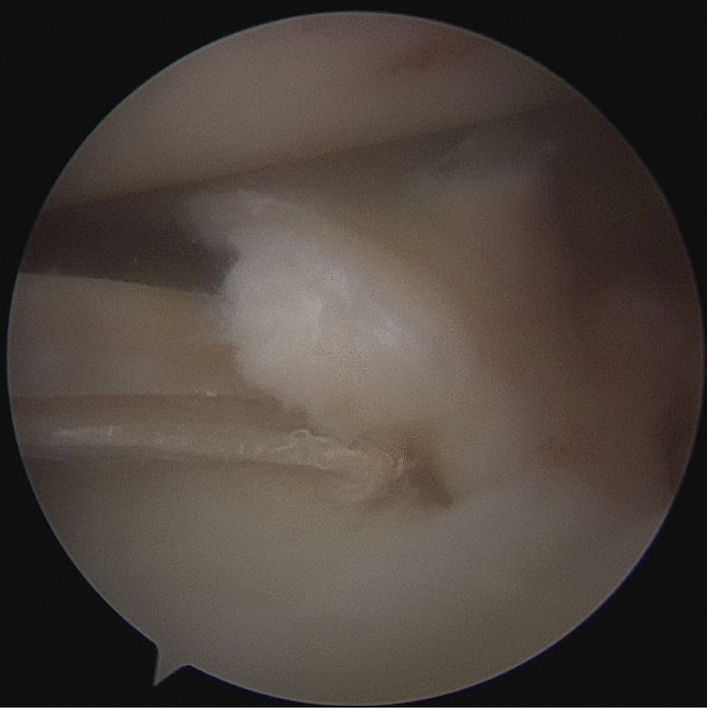
The gracilis tendon passage through the medial meniscus posterior root.

**Figure 5:**
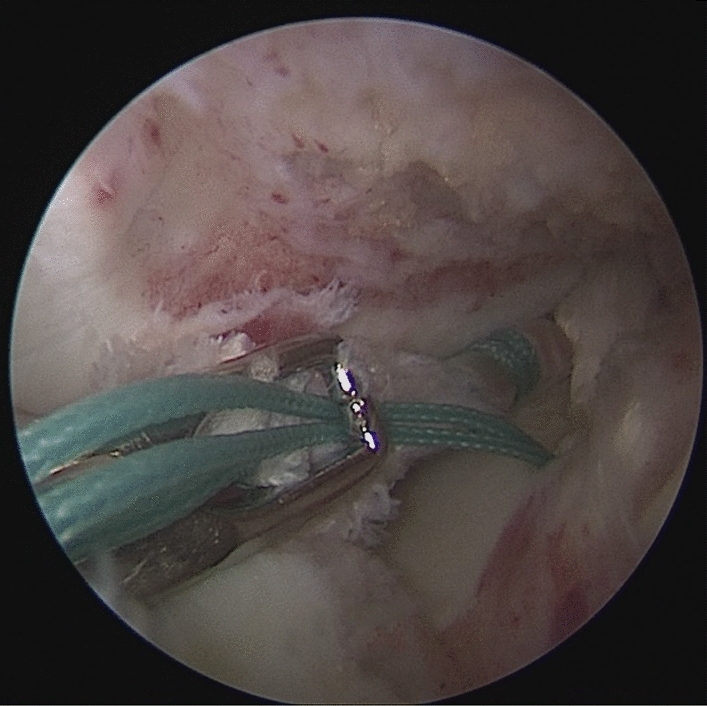
The gracilis tendon are shuttled into the tibial tunnel.

**Figure 6:**
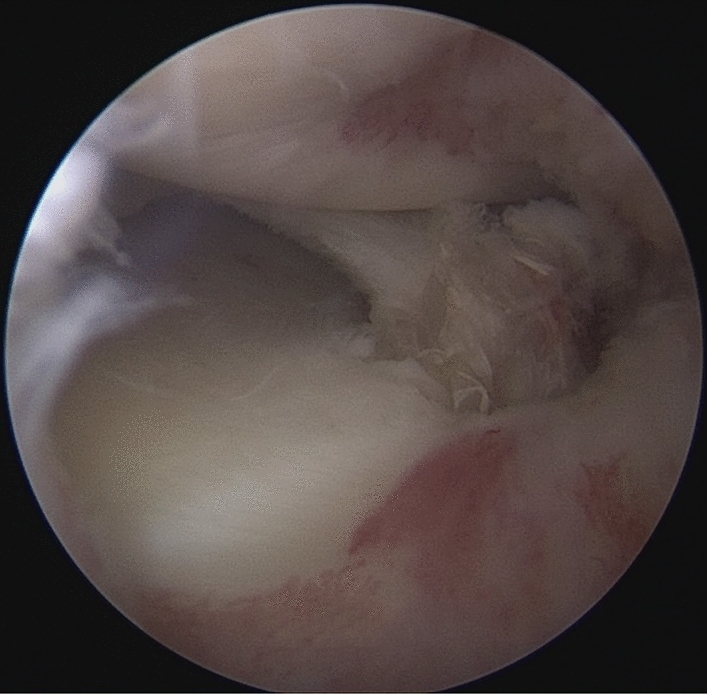
The arthroscopic visualization is used to maintain the appropriate position and tension of the graft.

Patients were placed in a supine position with knee flexion of 90°, and a pneumatic tourniquet was used after spinal anesthesia. The gracilis tendon was harvested via a 2 cm longitudinal incision over the pes anserinus and the graft was prepared and the ends were whipstitched with a No. 0 fiber wire. Firstly, the arthroscopic evaluation and treatment of the MMPRTs and other intraarticular lesions (Fig. 1). A limited refresh was applied to the torn edge of the meniscus with a motorized shaver, and a guide pin was drilled from a small incision over the anterior proximal tibia and advanced to the original anatomy of meniscus root under guide system assistance (Smith & Nephew), then the guide pins were over-drilled with a cannulated 6-mm drill. A self-passing suture device (Sharpshooter, Ivy Medical) was placed in the accessory portal to pass a No. 0 fiber wire suture (Smith & Nephew) through the posterior portion of the torn meniscus root (Fig. 2), then, the soft tissue tunnel is dilated with multiple passes of No. 0 fiber wire (Fig. 3) followed by the tendon graft passage through the medial meniscus posterior root (Fig. 4). Finally, tendon grafts are shuttled into tibial tunnel (Fig. 5) and its tails fixed with anchor (6-mm PEEK knotless suture anchor; Smith & Nephew) to tibia. Arthroscopic visualization is used to maintain the appropriate position and tension of the graft (Fig. 6).

### Postoperative management

Muscular strengthening exercises were started on the first postoperative day, including quadriceps strengthening exercises and straight leg lifts. Moreover, passive knee flexion was performed immediately after the surgery and gradually increased to 90° of flexion after 2 weeks, and progressed to full flexion of the knee at the end of 8 weeks. Patients were allowed to walk non-weight-bearing with two crutches for six weeks after surgery, and weight bearing was started progressively at 6 weeks as tolerated by the patient. Weight-resisted exercise and half squat exercise were permitted at least 12 weeks after surgery, and heavy strenuous activities and sports activities were started at 6 months postoperatively.

### Outcome assessment

All patients were followed-up regularly, the imaging and clinical examinations were performed preoperatively and postoperatively at 1 month, 3 months, 6 months, 1 year and yearly until final follow-up. The knee functional assessment consisted of evaluation done using VAS score, Lysholm score, and IKDC score^14^ evaluated by 2 orthopedic surgeons and performed before surgery and at final follow-up.

At the final follow-up, the orthopedic surgeon and radiologist were blinded to patients’ information and evaluated the healing status of the repaired MMPRTs based on knee MRI. Meniscal root healing status was assessed according to the criteria of previous studies^15^. Complete meniscal root healing was considered if the repaired MMPRT was in continuity with the bone on sagittal, coronal and axial images. Incomplete healing includeed partial healing (loss of continuity in at least 1 view on any one of the sagittal, axial or coronal images) and failed healing (no continuity and no evidence of meniscal healing at the repair site).

### Statistical analysis

Quantitative variables were presented as mean value ± standard deviation, and the two groups were compared using the Student’s t-test. Count variables were expressed as numbers and percentages and were assessed by the Chi-square test. Risk factors related to meniscus root healing status were analyzed by the binary logistic regression analysis model. The receiver operating characteristic (ROC) curve was used to assess the predictive value of risk factors related to incomplete healing. The sensitivity and specificity were calculated based upon optimal cut off scores, and accuracy was determined by the area under the curve (AUC). Statistical significance was set as a P value less than 0.05. All analysis was performed by IBM SPSS Version 22 (SPSS Inc. Chicago IL).

### Ethical approval and consent to participate

This study was approved by the ethics committee of the Jiangxi Provincial People’s Hospital (The First Affiliated Hospital of Nanchang Medical College). All procedures performed in this study involving human participants were in accordance with the bioethical standards of the institutional and national research committees and with the 1964 Declaration of Helsinki and its later amendments. Written informed consent was obtained from individual or guardian participants.

## Results

### Patient demographics

We conducted a retrospective review of medical records of 171 patients who received arthroscopically assisted tendon graft fixation of the MMPRTs between January 2018 and September 2021. However, 42 patients were excluded from the study (concomitant multiple ligament injuries: 14 cases; concomitant high tibial osteotomy: 21 cases; follow-up loss: 7 cases), ultimately, 129 MMPRTs patients who received arthroscopically assisted tendon graft fixation of the meniscal root by a single orthopaedic surgeon was recruited, including 98 patients in the complete healing group and 31 patients in the incomplete healing group (partial healing: 26 cases; and failed healing: 5 cases). As presented in Table 1, there were no significant differences in gender, injured side, K-L grade, comorbidities, MMPRTs type, follow-up time, and length of postoperative hospital stay among the two groups (P > 0.05). Moreover, intraoperative and postsurgical complications, such as deep venous thrombosis, infection, and knee stiffness were not found. Based on the analysis, the Age, BMI, preoperative meniscus extrusion and varus degree were significantly higher in the incomplete healing group patients than those patients in the complete healing group (p = 0.008, p = 0.001, p < 0.001, p < 0.001; respectively).

**Table 1:** The demographics of the patients with repaired MMPRTs.

Factors	Complete healing (98)	Incomplete healing (31)	P
Gender: male n (%)	52 (53.0%)	18 (58.1%)	0.792
Side, right (%)	57 (58.2%)	20 (64.5%)	0.675
Age (yr)	31.4 ± 10.4	45.7 ± 9.3	**< 0.001**
BMI (kg/m^2^)	22.6 ± 3.3	25.9 ± 5.1	**< 0.001**
Comorbidities: yes n(%)			
Diabetes mellitus	7 (7.1%)	6 (19.4%)	0.101
High blood pressure	12 (12.2%)	6 (19.4%)	0.395
Smoking status	14 (14.3%)	4 (12.9%)	1
Alcohol consumption status	12 (12.2%)	2 (6.5%)	0.522
K-L			0.434
0	38 (38.8%)	11 (35.5%)	
1	50 (51.0%)	14 (45.2%)	
2	10 (10.2%)	6 (19.4%)	
MMPRTs type			0.330
2	40 (10.1%)	8 (25.8%)	
3	18 (18.4%)	5 (16.1%)	
4	21 (21.4%)	8 (25.8%)	
5	19 (19.4%)	10 (32.3%)	
Follow-up time (months)	27.2 ± 2.6	27.2 ± 2.9	0.982
Preoperative meniscus extrusion (mm)	2.7 ± 1.2	4.1 ± 0.7	**< 0.001**
Varus degree (°)	2.2 ± 1.0	4.0 ± 0.9	**< 0.001**
Postoperative hospital stays (d)	3.5 ± 1.1	3.3 ± 1.0	0.217
Postoperative complications	0	0	1

MMPRTs: medial meniscal posterior root tears; BMI: body mass index; K-L: Kellgren-Lawrence.

### Risk factors correlated with meniscus root healing status in patients with repaired MMPRTs

Tables 2, 3 shows the functional recovery of the knee joint in the different groups. The postoperative pain in the knee was relieved in almost all cases, and the Lysholm score and IKDC score were significantly improved at the end of follow-up (P < 0.001; respectively). Furthermore, compared with the incomplete healing group, the complete healing group was significant improved in the VAS score, Lysholm score and IKDC score at final follow-up (P < 0.05; respectively).

**Table 2:** Functional results of the study groups.

Clinical outcome or score	Complete healing (98)	Incomplete healing (31)	P
Preoperative VAS score	5.3 ± 1.3	5.2 ± 1.3	0.566
Preoperative Lysholm score	58.2 ± 8.0	58.9 ± 7.4	0.617
Preoperative IKDC score	59.1 ± 7.7	58.4 ± 7.4	0.687
Final follow-up VAS score	1.0 ± 0.8	2.3 ± 0.9	** < 0.001**
Final follow-up Lysholm score	89.1 ± 7.0	75.1 ± 8.8	** < 0.001**
Final follow-up IKDC score	88.8 ± 6.0	75.5 ± 8.7	** < 0.001**

VAS: visual analogue scale; IKDC: international knee documentation committee score.

**Table 3:** Functional Results of the Study Groups.

Characteristic	Preoperative	Postoperative	P
Complete healing			
VAS score	5.3 ± 1.3	1.0 ± 0.8	< 0.001
Lysholm score	58.2 ± 8.0	89.1 ± 7.0	< 0.001
IKDC score	59.1 ± 7.7	88.8 ± 6.0	< 0.001
Incomplete healing			
VAS score	5.2 ± 1.3	2.3 ± 0.9	< 0.001
Lysholm score	58.9 ± 7.4	75.1 ± 8.8	< 0.001
IKDC score	58.4 ± 7.4	75.5 ± 8.7	< 0.001

VAS: visual analogue scale; IKDC: international knee documentation committee score.

**Figure 7:**
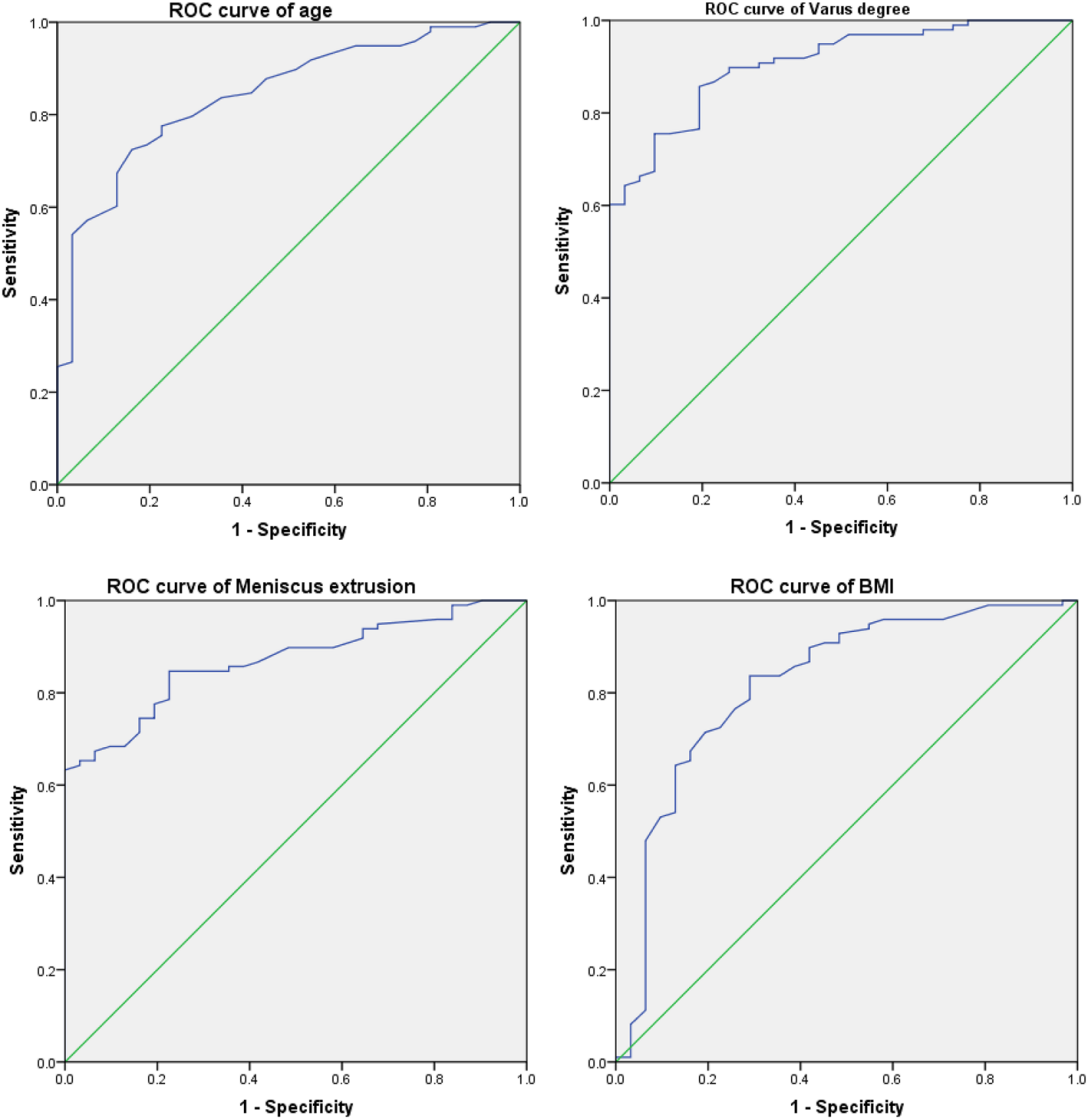
The receiver operating characteristic (ROC) curves of risk factors for predicting meniscus root healing status in patients with the arthroscopically assisted tendon graft fixation of MMPRTs. Age, ROC curve of age; BMI, ROC curve of BMI; meniscus extrusion, ROC curve of preoperative meniscus extrusion; varus degree, ROC curve of varus degree.

Binary logistic regression analysis was carried out to identify the independent risk factors related to incomplete healing (Table 4), and indicated that age (OR = 1.095, P = 0.039), BMI (OR = 1.259, P = 0.018), preoperative meniscus extrusion (OR = 5.181, P < 0.001) and varus degree (OR = 7.764, P < 0.001) were the independent risk factors correlated with incomplete healing in patients with repaired MMPRTs. Figure 7 and Table 5 display the risk factor values, with the varus degree exhibiting the highest predictive accuracy (AUC = 0.907; P < 0.001) for determining incomplete healing among these factors. It showed a sensitivity of 85.7% and specificity of 80.6%. The cut-off values for age, BMI, preoperative meniscus extrusion, and varus degree were determined as 37.5 years (Sensitivity: Specificity;), 24.5 kg/m^2^, 2.7 mm, and 3.3° respectively.

**Table 4:** Risk factors correlated with meniscus root healing status in patients with repaired MMPRTs.

Risk factor	odds ratio	95% confidence interval	P
Age (yr)	1.095	1.004–1.195	**0.039**
BMI (kg/m^2^)	1.259	1.040–1.524	**0.018**
Preoperative meniscus extrusion	5.181	1.819–14.752	** < 0.001**
Mechanical alignment, varus degree	7.764	2.501–24.104	** < 0.001**

MMPRTs: medial meniscal posterior root tears; BMI: body mass index.

**Table 5:** The diagnostic accuracy of risk factors correlated with meniscus root healing status.

Risk factors	Cut-off value	Sensitivity	Specificity	AUC	p
Age (yr)	37.5	72.4%	83.9%	0.843	** < 0.001**
BMI (kg/m^2^)	24.5	83.7%	71.0%	0.821	** < 0.001**
Meniscus extrusion(mm)	2.7	63.3%	100%	0.869	** < 0.001**
Varus degree (°)	3.3	85.7%	80.6%	0.907	** < 0.001**

BMI: body mass index; AUC: area under the curve.

## Discussion

The main finding of this study was that the complete meniscus root healing rates of patients who received arthroscopically assisted tendon graft fixation of MMPRTs was approximately 76.0%, and there was a significant improvement of VAS score, Lysholm score, and IKDC score postoperatively. Furthermore, we identified age > 37.5 years, BMI > 24.5 kg/m^2^, preoperative meniscus extrusion > 2.7 mm and varus degree > 3.3° as independent risk factors for incomplete meniscus root healing in patients who received arthroscopically assisted tendon graft fixation of MMPRTs. The MMPRTs result in higher contact pressures and decreased contact area, and strongly correlated with knee osteoarthritis^15^. Compared with partial meniscectomy, more evidence indicates that the anatomic fixation of MMPRTs can restore both the anatomy and hoop tension of the medial meniscus^16^. Transtibial pull-out repair by securing the meniscus root to its original anatomy has been reported as a promising approach to clinical improvement^17^. Li et al.^18^ demonstrated the successful use of semitendinosus tendon autograft in a rabbit model resulting in biomechanical properties that were similar to that of the normal meniscus. Additionally, compared to the transtibial pull-out technique, the arthroscopically assisted meniscus root reconstruction with gracilis autograft demonstrated advantages in treating patients, resulting in superior clinical outcomes and higher rates of meniscus root healing^19,20^. Like the anterior cruciate ligament model, the reinforced reconstruction technique that the initial fixation is strong enough to resist initial displacement and provide a long-term stability structure allows the tendon graft to heal the meniscus and bony tunnels^21^. Consistently with previous studies, our study yielded optimal results in complete meniscal root healing rate (76.0%) at postoperative MRI.

Seo et al.^22^ analyzed the outcomes of the transtibial pull-out repaired MMPRTs healing status according to second-look arthroscopy, and all the 11 knees showed incomplete meniscus healing at the final follow-up, nevertheless, the meniscus root healing status appeared to be unrelated to the clinical symptomatic improvement. On the other hand, Dragoo et al.^23^ demonstrated that the meniscal root healing status after arthroscopic all-inside repair seemed also associated to clinical symptomatic improvement: improved symptoms were obtained in all patients with complete meniscal root healing, and good clinical outcomes were detected in the incomplete healing group. The current study also showed that both the complete healing group and incomplete healing group can provide good clinical outcomes, even if the complete healing group had a significant difference in postoperative VAS, Lysholm, IKDC scores compared with incomplete healing group values. However, it is unclear why most patients showed improved clinical outcomes after MMPRTs were repaired by the arthroscopically assisted tendon graft fixation of the meniscus root, even though the structural integrity of the repaired meniscus root was not observed on knee MRI.

Recently, MMPRTs have become increasingly recognized, but the risk factors correlated with the meniscal root healing status of repaired MMPRTs have not been elucidated in the literature. Hwang et al.^24^ investigated 476 consecutive patients who received an arthroscopic procedure on their medial meniscus, and found that the persons with MMPRTs had significantly increased age, higher BMI and greater varus mechanical axis angle compared with persons with other types of the meniscal tear. Chung et al.^25^ investigated 40 patients who were followed for > 5 years after pullout fixation of MMPRTs, and indicated that older age was one of the risk factors to predict a poor prognosis. In our study, the results showed that age was correlated with meniscus healing status, with a relatively low cut off value of 37.5 years old.

Increased knee compartment pressures as a result of higher BMI could impart increased strain and rotational stress in the knee joint, which may potentially explain the high frequency of meniscus tears in these patients. Additionally, Obesity increases lower extremities pressure being nonetheless strongly correlated with the development of cartilage degeneration and osteoarthritis. Previous studies have shown that MMPRTs are more likely to be associated with higher BMI compared to other types of medial meniscal tear^24^. Furthermore, significant differences in BMI were observed between patients with and without poor clinical outcomes after undergoing pullout repair of MMPRTs in well-aligned knees. Moreover, Zhang et al.^26^ showed that BMI > 30 kg/m^2^ was a risk factor for unfavorable clinical outcomes. Interestingly, our study indicated that BMI was an independent risk factor correlated with meniscus root healing status. The cutoff value of it was 24.5 kg/m^2^, and the sensitivity and specificity were 83.7% and 71.0%, which suggests that the arthroscopically assisted tendon graft fixation of MMPRTs should be carefully considered in patients with higher BMI.

Meniscus extrusion could also affect the clinical outcome of repaired MMPRTs, compared with the increased meniscus extrusion patients at 1 year, in fact Chung et al.^27^. showed that patient with decreased meniscus extrusion have more favorable clinical scores (final Lysholm and IKDC scores in decreased meniscus extrusion was 88.1 ± 12.1 and 79.0 ± 11.4 vs. increased extrusion was 81.0 ± 9.0 and 71.1 ± 7.8) and radiographic findings, thus, a significant correction of extrusion is determinable to protective knee function and the load transmission. Chung et al.^28^ investigated 37 patients who underwent MMPRTs pull-out repair with followed 10 years, and indicated that in patients with meniscal extrusion of more than 0.7 mm, increased extrusion was associated to higher risk of clinical failure. The current study also showed that preoperative meniscus extrusion is a significant risk factor correlated with meniscus root healing status after the arthroscopically assisted tendon graft fixation of MMPRTs. Furthermore, the current study determined that the cut-off value of preoperative meniscus extrusion which can lead to incomplete meniscus root healing is 2.7 mm.

Increased varus alignment which potentially leads to increased lower extremity pressure and is strongly correlated with the development of osteoarthritis, has been considered an important predicting factor correlated with unfavorable clinical outcomes in several fields^29^. McCormick et al.^30^ investigated 172 patients who were followed for > 2 years after meniscus allograft transplantation, and confirmed that preoperative varus alignment played a significant role in the longer allograft survival and favorable outcomes. Moreover, Chung et al.^28^ confirmed that preoperative varus alignment is a risk factor for clinical failure of repaired MMPRTs in a long-term perspective with a threshold identified at preoperative varus alignment of 5 degrees (sensitivity of 75% and specificity of 76%), above which the failure risk would be significantly increased. This is because varus alignment tends to exert increased stress and pressures in the medial compartment of the repaired knee, which may potentially influence the healing of the fixed meniscus root. Consistently with previous studies, our study indicated that varus alignment is an independent risk factor correlated with meniscus root healing status with a cut-off value of 3.3 degrees (sensitivity 85.7%, specificity 80.6%).

Several limitations were also detected in this study. First, our current study is a single-center study, and a relatively small number of patients and a non-randomized comparative retrospective study may introduce bias into the results. Second, we just observed the knee functional scores and meniscus root healing status within 2 years after medial meniscal root reconstruction, the follow-up period is relatively short and no second-look arthroscopy has been performed. Given that postoperative meniscus root healing status is generally assessed at postoperative 2 years, these findings must be considered valuable information.

## Conclusions

Based on results from the current study, the arthroscopically assisted tendon graft fixation of MMPRTs can provide good clinical and radiological outcomes. Additionally, we identified age > 37.5 years, BMI > 24.5 kg/m^2^, preoperative meniscus extrusion > 2.7 mm and varus degree > 3.3° as independent risk factors correlated with incomplete meniscus root healing status. Thus, these negative predicting factors should be taken into consideration before performing root repair in patients with MMPRTs.

## Data Availability

All the data will be available upon reasonable request to the corresponding author of the present paper.
